# Evaluating a Process for Offering Psychiatry Inpatients a Novel Onsite Sexual and Reproductive Health Clinic

**DOI:** 10.1192/bjo.2024.399

**Published:** 2024-08-01

**Authors:** Francesca Moss-Lawton, Anushka Pathak, Emily Giles, Norman Nuttall, Nicole Needham

**Affiliations:** NHS Lothian, Edinburgh, United Kingdom

## Abstract

**Aims:**

1. For all eligible general adult psychiatry and substance misuse inpatients at the Royal Edinburgh Hospital (REH) to be offered appointments at a pilot onsite sexual and reproductive health (SRH) clinic.
2. To evaluate the need for this novel service using eligibility rates and attendance levels.

**Methods:**

Eligibility of all inpatients on a substance misuse ward considered at admission, and a space in the clinic offered if appropriate. Reminder added to the clerking proforma.

Eligibility of general adult psychiatry (GAP) inpatients considered by their multidisciplinary team (MDT) weekly. Team 1 to trial this at ward round, and team 2 to trial it at rapid rundown.

A patient leaflet was created to explain the clinic.

**Results:**

**General adult psychiatry:** In team 1, 82% (120/147) of patients were considered by the MDT over 20 weeks, and in team 2, 65% (53/82) over 10 weeks. Of all GAP patients considered, 48% (83/173) were deemed eligible. Of those, 70% (61/83) were asked if they wished to attend, usually by the junior doctor leading the QI project. Thirty-six percent (22/61) of those booked into the clinic, of which 82% attended.

**Substance misuse ward**: Over 15 weeks, 85% (82/97) of patients admitted to the substance misuse ward were considered, deemed eligible and offered a space in the clinic at admission, of whom 15 accepted and 4 attended.

**Conclusion:**

Nearly half of GAP inpatients were eligible to attend, with the total likely to be higher over time, as mental state improved. A high level of demand was demonstrated for SRH services in this population, where research also suggests a higher level of need and lower levels of access.

During weeks when the QI leads were absent, it was not recorded that any patients were considered at ward rounds or rapid rundowns. It was difficult to implement a process for this whilst the clinic was still at the pilot stage. The incorporation of a reminder into the ward round template would ensure that this is always considered.

A very high proportion of substance misuse patients were eligible for this clinic, highlighting higher levels of capacity. The main challenges for attendance were a high discharge rate, presence of withdrawal symptoms, and extensive passes off the ward.

